# Role of Socioeconomic Status in National Health Insurance Ownership in Indonesia’s Rural Areas

**DOI:** 10.34172/jrhs.2024.143

**Published:** 2024-03-18

**Authors:** Agung Dwi Laksono, Ratna Dwi Wulandari, Diyan Ermawan Efendi, Tumaji Tumaji, Zainul Khaqiqi Nantabah

**Affiliations:** ^1^National Research and Innovation Agency Republic of Indonesia, Jakarta, Indonesia; ^2^Faculty of Public Health, Universitas Airlangga, Surabaya, Indonesia

**Keywords:** National health program, Socioeconomic disparities in health, Economic status, Public health

## Abstract

**Background:** National Health Insurance (NHI) is one of the Indonesian Government’s policies to increase public access to health services. The study analyzed the role of socioeconomic status in NHI ownership in rural Indonesia.

**Study Design:** A cross-sectional study.

**Methods:** The study population included residents of rural Indonesia. The study examined secondary data from the survey entitled "Abilities and Willingness to Pay, Fee, and Participant Satisfaction in Implementing National Health Insurance in Indonesia in 2019", involving 33225 respondents representing Indonesia’s rural areas. The study was conducted from March to December 2019. The variables analyzed included NHI, socioeconomic level, age group, gender, education level, employment status, and marital status. In the final step, the study employed binary logistic regression to explain the relationship between socioeconomic status and NHI ownership.

**Results:** The results show that 63.8% of the population in rural Indonesia participated in the NHI. The poorer residents were 1.235 times more likely to have NHI than the most impoverished population (AOR 1.235; 95% CI 1.234-1.237). People with middle wealth status were 1.086 times more likely to have NHI than the poorest (AOR 1.086; 95% CI 1.085-1.087). The richer residents were 1.134 times more likely to have NHI than the poorest (AOR 1.134; 95% CI 1.133-1.136). The richest residents were 1.078 times more likely to have NHI than the poorest residents (AOR 1.078; 95% CI 1.077-1.079).

**Conclusion:** The study concluded that socioeconomic status is related to NHI ownership in rural Indonesia. The analysis indicated that all socioeconomic categories were more likely to become NHI participants than the poorest in Indonesia.

## Background

 Health is one of the fundamental rights of all levels of society. Therefore, the government is obliged to provide adequate health services. The condition was stated in the 1945 Indonesian Constitution article 28 H paragraph 1 and article 34 paragraph 3.^1^ Meanwhile, the World Health Organization (WHO) also noted that the provision of adequate health services is the responsibility of a country’s government.^2^ Health services are acceptable with sufficient health facilities, easy access, and good service quality. The minimum standard in health services needs to be monitored continuously so that the community can get the best health services, especially with the geographical condition of Indonesia, which is an archipelagic country.^3,4^ The difficulty of geographical conditions does not eliminate the right of the community to obtain the same health services wherever they are; as stated in Article 25 of the Universal Declaration of Human Rights, the right to health services and social services must be equal.^5^ Therefore, the government must ensure that the community can easily access health services.

 One way to increase access to health services is to have health insurance. However, not all people can afford to register and have health insurance. One of the influencing factors is socioeconomic status. Research in Ghana shows that people from the poorer and poorest groups are less likely to have insurance because of the inability to pay premiums.^6^ The reason is uncertain income, considering that most lower-middle-class people work in the informal sector.^7,8^

 On the other hand, wealth status can increase the likelihood of enrolling in a national health insurance (NHI) scheme.^9^ A systematic review of 48 articles from 17 lower-middle-income countries showed that households from the wealthiest group had greater odds of applying for health insurance than the poorest group.^10^ Meanwhile, a study conducted in Jordan reported that the richest were 1.411 times more likely than the poorest to have health insurance (95% CI 1.145-1.737).^11^ Increased income was reported to be correlated with a higher willingness to pay health insurance premiums.^12^ Another factor that influences is the place of residence, and the percentage of health insurance ownership is much lower in rural areas than in urban areas.^9^

 Moreover, there are still gaps in the utilization of health services, especially between urban and rural areas. Rural communities use health services less due to poor access.^13,14^ Conversely, people in urban areas tend to have better access to health services because the private sector also provides health services.^15^ Unsurprisingly, people in urban areas are more likely to use health services than people in rural areas.^15,16^ In addition to the access factor, the selection of healthcare facilities is also influenced by the level of expenditure. The results of previous studies showed that there were gaps between different socioeconomic groups in utilizing health services.^17^ The health service utilization gap can be reduced by the ownership of health insurance, which has been proven to increase health service utilization.^18^

 The Indonesian government introduced the NHI in 2014. The program aims to fulfill the basic needs of the community in the health sector to protect the community.^8^ In this program, all people are required to become NHI participants. By participating, it is hoped that the community will be protected and get health service guarantees. However, it seems that not all people are registered as NHI participants. Based on records, of the approximately 275.7 million Indonesians, 242.6 million people have been registered as NHI participants until July 2022.^19^ In other words, 33.1 million people, or 12.01% of the total population, still have not become NHI participants. The study analyzed the role of socioeconomic status in NHI ownership in rural Indonesia.

## Materials and Methods

###  Data source

 This cross-sectional study employed secondary data from the survey entitled “Abilities and Willingness to Pay, Fee, and Participant Satisfaction in Implementing National Health Insurance in Indonesia in 2019.” The Ministry of Health of the Republic of Indonesia conducted the national survey. A total of 33 225 people were included in the study through multistage random sampling to represent Indonesia’s rural areas.

 Multistage cluster sampling is the sample design, which involves choosing multiple sub-districts in each region using Probability Proportional to Size (PPS) sampling, which takes into account the size of the number of households. Each province’s allocation of the chosen sub-districts is decided upon first. Using PPS sampling, two villages are chosen based on the size of the number of houses and families in each subdistrict. Each village uses systematic sampling, whereby the number of households/families is implicitly stratified, to choose one neighborhood. The goal of this is to guarantee sample proportionality. Subsequently, ten houses are chosen through systematic sampling from each neighborhood. Based on the sample design that is created subsequently, the sample frame is distinguished. Lists of subdistricts in each region, lists of villages/subdistricts in particular subdistricts, lists of neighborhoods in particular villages/subdistricts, and lists of households in particular neighborhoods are the four types.

###  Variables

 The study used NHI ownership as the outcome variable. NHI participant refers to the respondent’s involvement in the NHI, whether it is mandatory (civil servant, police, army), borne by the company, or recipient of contribution assistance from the government. NHI ownership consists of two categories: No (0) and Yes (1).

 The study employed socioeconomic status as the exposure variable. The research determined socioeconomic status based on the wealth index formula. Meanwhile, the wealth index was a composite measure of the total expenditure of a household. The study calculated the wealth index using easy-to-collect data on a household’s expenditure on health care insurance, food, housing, and other items. Moreover, the wealth index was divided into five categories: the poorest, poorer, middle, richer, and richest.^20,21^

 The study employed five other variables as control variables. These variables included age, gender, education level, employment status, and marital status. Furthermore, the age group was divided into three categories: ≤ 17, 18-64, and ≥ 65. Gender consisted of two categories: male and female, and the education level consisted of four categories: no education, primary, secondary, and higher. Meanwhile, employment status was divided into three categories: unemployed, formal, and informal. Moreover, marital status consisted of never married, married, and divorced/widowed.

###  Data analysis

 A bivariate analysis was conducted using the chi-square test in the initial stage. Furthermore, a collinearity test was conducted to ensure the absence of a strong correlation between independent variables in the resulting regression model. In the final stage, binary logistic regression was used. The multivariate relationship of exposure and control variables with NHI ownership as the outcome variable was tested. The adjusted odds ratios (AORs) were shown with 95% confidence intervals (CIs). The statistical analysis was performed using the IBM SPSS version 26.0.

## Results

 The results of the study indicated that 63.8% of the population in rural Indonesia participated in the NHI program. The rest were uninsured or had other health insurance. Table 1 reports the descriptive statistics of NHI ownership in rural Indonesia. The poorest respondents dominated the NHI group based on socioeconomic status. Meanwhile, male respondents dominated the NHI group.

**Table 1:** Descriptive statistic of national health insurance ownership (no/yes) in rural Indonesia (n = 33 225)

**Characteristics**	**No, n=12083**	**Yes, n=21142**	***P *** **value**
Socioeconomic status			0.001
Poorest	2937	4,743	
Poorer	2555	4,824	
Middle	2531	4,289	
Richer	2216	3,818	
Richest	1844	3,468	
Gender			0.001
Male	6106	10,635	
Female	5977	10,507	
Age group (y)			0.001
≤ 17	4276	5,662	
18-64	7137	14,105	
≥ 65	670	1,375	
Education level			0.001
No education	2672	2,998	
Primary	7138	12,907	
Secondary	2004	4,237	
Higher	269	1,000	
Employment status			0.001
Unemployed	6804	10,728	
Formal	456	1,991	
Informal	4823	8,423	
Marital status			0.001
Never married	5677	8,726	
Married	5729	11,086	
Divorced/widowed	677	1,330	

 Considering the age group, respondents aged 18-64 dominated the NHI category. Meanwhile, respondents with primary education outnumbered other groups in the NHI. Regarding employment status, unemployed respondents dominated the NHI group. Finally, married respondents dominated the NHI group.

 The results of the collinearity test indicated that there was no strong relationship between the independent variables. The tolerance value for all variables was more potent than 0.10. Additionally, the variance inflation factor (VIF)value for all variables was less than 10.00. The regression model exhibited no multicollinearity.

Table 2 shows the binary logistic regression of NHI ownership in rural Indonesia. The result shows that poorer people were 1.235 times more likely to have NHI than the most impoverished people (AOR 1.235; 95% CI 1.234-1.237). People with middle wealth status were 1.086 times more likely to have NHI than the poorest residents (AOR 1.086; 95% CI 1.085-1.087). On the other hand, the richer residents were 1.134 times more likely to have NHI than the poorest residents (AOR 1.134; 95% CI 1.133-1.136). Moreover, the wealthiest residents were 1.078 times more likely to have NHI than the most impoverished population (AOR 1.078; 95% CI 1.077-1.079). This analysis showed that all socioeconomic categories were more likely to become NHI participants than the poorest in Indonesia.

**Table 2:** The result of binary logistic regression of factors associated with national health insurance ownership in rural Indonesia (n = 33 225)

**Predictors**	**Adjusted OR (95% CI)**	***P *** **value**
Socioeconomic status		
Poorest		
Poorer	1.235 (1.234, 1.237)	0.001
Middle	1.086 (1.085, 1.087)	0.001
Richer	1.134 (1.133, 1.136)	0.001
Richest	1.078 (1.077, 1.079)	0.001
Gender		
Male	-	
Female	1.019 (1.018, 1.019)	0.001
Age group (y)		
≤ 17	-	
18-64	1.295 (1.293, 1.296)	0.001
≥ 65	1.529 (1.526, 1.532)	0.001
Educational status		
No education	-	
Primary	1.500 (1.499, 1.502)	0.001
Secondary	1.519 (1.517, 1.521)	0.001
Higher	2.019 (2.014, 2.023)	0.001
Employment status		
Unemployed	-	
Formal	2.037 (2.033, 2.040)	0.001
Informal	0.903 (0.902, 0.904)	0.001
Marital status		
Never married	-	
Married	0.959 (0.958, 0.960)	0.001
Divorced/widowed	1.060 (1.058, 1.062)	0.001

AOR: adjusted odds ratio; CI: confidence interval.

 Apart from Socioeconomics, the analysis results also found five other predictors related to NHI ownership. Considering gender, female participants had 1.019 times higher probability of having NHI than male participants (AOR 1.019; 95% CI 1.018-1.019). On the other hand, considering the age group, residents aged 18-64 were 1.295 times more likely to have NHI than residents in the ≤ 17 age group (AOR 1.295; 95% CI 1.293-1.296). Moreover, residents in the ≥ 65 age group were 1.529 times more likely to have NHI than residents in the ≤ 17 age group (AOR 1.529; 95% CI 1.526-1.532).

 Regarding education, people with a primary education level were 1.500 times more likely to have NHI than those without education (AOR 1.500; 95% CI 1.499-1.502). Residents with secondary education were 1.519 times more likely to have NHI than non-educated residents (AOR 1.519; 95% CI 1.517-1.521). Residents with higher levels of education were 2019 times more likely to have NHI than residents without education (AOR 2.019; 95% CI 2.014-2.023). This information shows that the higher the level of education, the higher the opportunity for residents in rural Indonesia to have an NHI.

Table 2 shows that people with formal work were 2.037 times more likely to have NHI than the unemployed (AOR 2.037; 95% CI 2.033-2.040). Residents with the informal type of work were 0.903 times less likely to have NHI than the unemployed residents (AOR 0.903; 95% CI 0.902-0.904). Meanwhile, married residents were 0.959 times less likely to have NHI than never married residents (AOR 0.959; 95% CI 0.958-0.960). Moreover, divorced/widowed residents were 1.060 times more likely to have NHI than those who have never been married (AOR 1.060; 95% CI 1.058-1.062).

## Discussion

 This study demonstrated a significant positive association between health insurance ownership and wealth status. The odds were generally higher among respondents with all wealth statuses than the poorest households. This finding is in line with earlier studies conducted in Jordan, sub-Saharan African countries, and Indonesia that showed a positive association between health insurance ownership and economic status.^9,22^ This finding, however, underscores the importance of paying attention to the population who live with financial hardship, as they are most likely to have difficulties accessing quality health services.^23,24^

 Previously, the government had released the contribution assistance recipients (CAR) policy in the NHI to overcome the barrier to access health financing for people experiencing poverty.^25^ The Indonesian National Social Security Council reports that the national NHI membership is 86.96%, the non-contribution assistance recipient (NAR) is 40.4%, and the CAR is 59.6% based on the integrated monitoring and evaluation system.^26^ According to the membership segment, the government reports the following participation rates up to December 2021: workers getting 25.5% of earnings, non-wage recipients 13.1%, non-workers 1.9%, central CAR 42.4%, and regional CAR 17.1%.^26^

 This study also detected a significant positive association between the female gender and the possession of health insurance in Indonesia. This result might be because women need more healthcare services in their lives than men. For instance, most women would at least give birth once, which men would never experience in their lifespan, and thus higher expenses related to healthcare are borne by women.^27-30^ The ownership of health insurance is one of the ways to protect women from financial catastrophe. This finding confirms the result of a previous study conducted in Kenya that reported higher odds of health insurance ownership among women than men.^31^

 The result of binary logistic regression revealed that the odds of having health insurance rise as the age increases. It is assumed that as people age, their health deteriorates faster compared to younger people. As a result, older people are more inclined to enroll in health insurance to protect themselves against the increased chance of sickness.^32-34^ Besides, this result may be explained by the fact that financial stability grows with age, increasing one’s ability to obtain health insurance. This finding corroborates the results of studies conducted in Kenya and Ghana.^35,36^

 Another critical factor significantly associated with health insurance ownership in Indonesia’s rural areas was the education level. This study indicates that people with higher education have higher odds of having health insurance. High education enables people to understand their health needs, anticipate health-related expenditures, and make informed decisions. People with good education are most likely to have well-paid jobs that allow them to pay for health insurance.^7^ This result is consistent with studies conducted in other countries.^7,9^

 Interestingly, working in the informal sector showed a significant inverse association with health insurance ownership. One possible explanation for this circumstance is that the monthly regular health insurance premium was regarded as incompatible given the volatility of informal sector revenues. Income uncertainty and limited economic opportunities in rural areas of Indonesia might have hindered the ability of the people to pay the health insurance premium and thus lowered the ownership coverage.^37,38^ Previous studies carried out in Indonesia and Bangladesh revealed the difficulties of the informal sector workers in affording health insurance.^27,38^ The previous research has also demonstrated that, unlike the formal sector, assessing income and collecting income taxes from employees in the informal sector is challenging.^32^

 On the other hand, working in the formal sector is positively associated with health insurance ownership. Unlike the informal sector workers, the formal sector workers have stable monthly incomes that enable them to pay the insurance premium. Besides, most formal institutions in Indonesia oblige their employees to have health insurance. This finding corroborates the results of a study in Kenya.^32^

 The results of multivariate analysis indicated that marital status was significantly associated with the ownership of health insurance; in other words, the divorced/widowed respondents were more likely to have health insurance. Since people who are formerly married or have been married are generally in adult and old age groups, they are most likely to be financially stable. They have a good attitude toward health insurance.^9,22,39^ On the other hand, the married status was found to be negatively associated with health insurance ownership. This finding contrasts with the results of studies conducted in Kenya, Ghana, Zambia, and Togo.^7,32,36,40^ One possible explanation for this circumstance is that the Indonesian NHI provisions require the head of the family to bear the premium for all family members. Therefore, the bigger the family size, the higher the expenditure related to health insurance.

 This study examined a large amount of data to represent information on a national scale. On the other hand, this study analyzed secondary data; therefore, the authors evaluated variables that were limited to accepted ones. Several other characteristics connected to health insurance ownership that have been identified in earlier studies, such as income, smoking behavior, and a history of chronic disease, were not investigated.^27,37,41^

**Highlights**

- The study analyzes the role of socioeconomic status in national health insurance ownership in rural Indonesia.
- The study concluded that socioeconomics is related to NHI ownership in rural Indonesia.
- The analysis indicated that all socioeconomic categories were more likely to become NHI participants than the poorest in Indonesia.

## Conclusion

 Based on the results, it can be concluded that socioeconomics is related to NHI ownership in rural Indonesia. All socioeconomic statuses are more likely to have an NHI than the poorest. The results of this study indicate that the Indonesian government still has an extensive range of responsibilities to address the socioeconomic disparity. The government’s policy of releasing the CAR to address this situation still needs to be improved.

## Acknowledgments

 The authors would like to thank the Ministry of Health of the Republic of Indonesia for permission to analyze the data from the survey entitled “Abilities and Willingness to Pay, Fee, and Participant Satisfaction in the Implementation of NHI in Indonesia in 2019” in this article.

## Authors’ Contribution

**Conceptualization:** Agung Dwi Laksono, Ratna Dwi Wulandari.

**Data curation:** Ratna Dwi Wulandari.

**Formal analysis:** Agung Dwi Laksono.

**Funding acquisition:** Ratna Dwi Wulandari.

**Investigation: **Diyan Ermawan Effendi, Tumaji Tumaji, Zainul Khaqiqi Nantabah.

**Methodology:** Agung Dwi Laksono, Ratna Dwi Wulandari.

**Project administration:** Ratna Dwi Wulandari.

**Resources:** Tumaji Tumaji.

**Software:** Zainul Khaqiqi Nantabah.

**Supervision:** Agung Dwi Laksono.

**Validation:** Ratna Dwi Wulandari.

**Visualization:** Diyan Ermawan Effendi.

**Writing–original draft:** Ratna Dwi Wulandari, Diyan Ermawan Effendi, Tumaji Tumaji, Zainul Khaqiqi Nantabah.

**Writing–review & editing:** Agung Dwi Laksono.

## Competing Interests

 The authors declare that they have no competing interests.

## Ethical Approval

 The study employed secondary data. The National Ethics Commission classified this study as “excepted”. Moreover, the National Ethics Committee of the National Institute for Health Research and Development has approved the Ethical clearance of the survey entitled “Abilities and Willingness to Pay, Fee, and Participant Satisfaction in the Implementation of NHI in Indonesia in 2019” (Number: LB.0201/2/KE.340/2019). The names of the respondents were removed from the dataset used in the study. Written informed consent was obtained from all subjects or their legal guardians.Moreover,all methods were conducted following relevant guidelines and regulations.

## Funding

 Self-funded.
